# Uniform Molecular Alignment on Ag-Doped Nickel Oxide Films

**DOI:** 10.3390/nano15060449

**Published:** 2025-03-15

**Authors:** Dong Wook Lee, Tae-Hyun Kim, Young Kwon Kim, Dae-Shik Seo

**Affiliations:** 1 Department of Electrical and Electronic Engineering, Jeonju University, 303 Cheonjam-ro, Wansan-gu, Jeonju-si 55069, Jeollabuk-do, Republic of Korea; apxmfptl@naver.com; 2 Energy Industry Promotion Group, Jeonbuk Technopark, 110-5 Ballyong-ro, Deokjin-gu, Jeonju-si 54853, Jeollabuk-do, Republic of Korea; ygkim@jbtp.or.kr; 3 IT Nano Electronic Device Laboratory, Department of Electrical and Electronic Engineering, Yonsei University, 50 Yonsei-ro, Seodaemun-gu, Seoul 03722, Republic of Korea

**Keywords:** Ag nanoparticle, Ag-doped nickel oxide film, anisotropic structure, liquid crystal improved alignment, hysteresis reduction

## Abstract

This study presents the uniform alignment of liquid crystal (LC) molecules on silver (Ag)-doped nickel oxide (NiO) films. The films were fabricated using a solution brush coating process, with Ag doping concentrations of 0, 10, and 20 wt%. X-ray photoelectron spectroscopy confirmed the successful formation of the films, while atomic force microscopy revealed nano/microgroove anisotropic structures, attributed to brush hair movement during coating. X-ray diffraction analysis indicated the films’ amorphous nature. Optical transmittance measurements demonstrated their suitability for electronic display applications. Polarized optical microscopy verified uniform LC molecular alignment and effective optical control. The fabricated LC cells exhibited increased LC polar anchoring energy, improving device stability. The polar anchoring energy increased by 1159.02% after Ag doping. Additionally, reduced residual charge was observed, suggesting minimized image sticking. These findings indicate that Ag-doped NiO films are a promising alternative for LC alignment layers in functional LC systems.

## 1. Introduction

The rapid advancement of display technologies, including augmented reality, virtual reality, and functional electronics, has garnered significant attention in recent years. Liquid crystal displays (LCDs) have remained a focal point due to their exceptional durability, high resolution, and superior electro-optical properties [1,2,3,4].

Liquid crystals (LCs), the core materials in LCDs, exhibit unique characteristics that position them between liquid and solid states. They possess anisotropic properties in refractive index and dielectric constant, along with elastic and collective behaviors [5,6,7]. These properties make LCs particularly suitable for electronic display applications, where precise optical modulation is required.

In LCDs, the alignment layer plays a crucial role in determining the initial molecular orientation of LCs, ensuring uniform alignment essential for stable and high-quality display performance. This layer enables effective screen control in response to applied voltages, meeting the demands of modern electronic displays [8,9]. Traditionally, polyimide (PI) films processed via the rubbing method have been widely used for LC alignment. However, this approach presents several challenges, including contamination, local defects, and electrostatic damage. To overcome these issues, alternative alignment techniques such as evaporation, electron beam lithography, plasma treatment, sputtering [10,11,12,13,14,15], and brush coating [16,17,18] have been extensively explored.

Most of these alignment processes require a multi-step procedure involving film deposition followed by surface treatment. In contrast, the brush coating method offers a significant advantage in process efficiency, as it enables simultaneous film deposition and alignment layer formation. During brush coating, shear stress generated by the moving brush hairs induces nanoscale molecular alignment, similar to the frictional effects that create anisotropic structures in conventional rubbing methods [16,19]. As a solution-based approach, brush coating can also be integrated with the sol-gel method, a highly efficient technique for forming metal-inorganic oxide films.

In this study, nickel oxide (NiO) was selected as the alignment layer due to its favorable dielectric properties, making it a promising candidate for electro-optical applications in electronic displays [20]. Research related to the electrical properties of NiO has recently attracted much attention [21,22]. Research on doped films for enhancing film characteristics has recently attracted much attention from researchers [23,24]. In addition to their applicability during the fabrication of the alignment layer, silver (Ag) nanoparticles offer advantages in terms of optical properties and electrical conductivity; for this reason, NiO was doped with Ag nanoparticles [25,26]. The LC molecular alignment was analyzed using polarized optical microscopy (POM). Optical transmittance of the Ag-doped NiO films was evaluated using ultraviolet–visible–near-infrared (UV-Vis-NIR) spectroscopy. Surface morphology was characterized by atomic force microscopy (AFM) and transmission electron microscopy (TEM), while crystallinity was assessed using X-ray diffraction (XRD). The stoichiometric composition was examined via X-ray photoelectron spectroscopy (XPS). Finally, the electro-optical properties of the Ag-doped NiO films were investigated through LC polar anchoring energy and hysteresis measurements. These analyses aimed to evaluate the potential of Ag-doped NiO films as an alternative LC alignment layer, emphasizing their electro-optical performance and suitability for advanced display technologies.

## 2. Materials and Methods

Ag-doped NiO films were fabricated on 2 × 3 cm glass substrates. The substrates were cleaned using acetone and isopropyl alcohol, followed by ultrasonic treatment for 10 min in each solvent to eliminate impurities. Afterward, the substrates were dried with nitrogen gas. A 0.1 M solution of nickel (II) chloride hydrate was prepared in 2-methoxyethanol, with Ag nanopowder (99.5% trace metals basis) doped at concentrations of 0, 10, and 20 wt%. The Ag nanopowder was purchased from Sigma-Aldrich. The solution was stirred at 420 rpm at 75 °C for 2 h, then aged for 1 d to ensure stability. The brush hairs were thoroughly wetted with the solution, and the substrates were coated by moving the brush in a single direction. A thermal curing process was performed at 230 °C for 2 h, resulting in the final Ag-doped NiO films.

The surface morphology of the Ag-doped NiO films was analyzed using TEM (JEM-F200, JEOL, Tokyo, Japan) at an accelerating voltage of 200 kV, as well as AFM (NX-10, Park Systems, Suwon, Republic of Korea), with corresponding line-profile data. The crystalline structure of the NiO films was investigated using XRD (DMAX-IIIA, Rigaku, Tokyo, Japan) with a 2-theta scan over an angular range of 20–80°. The chemical composition of the films was analyzed using XPS (K-alpha, Thermo Scientific, Waltham, MA, USA), utilizing a monochromatic Al X-ray source (Al Ka line: 1486.6 eV) at 12 kV/3 mA. Optical transmittance was measured using UV-Vis-NIR spectroscopy (JASCO Corporation, V-650, Tokyo, Japan) in the wavelength range of 250–850 nm.

Antiparallel (AP) LC cells were assembled with Ag-doped NiO films to investigate the LC molecular alignment. Positive nematic LCs (IAN-5000XX T14, Δn = 0.111, ne = 1.595, no = 1.484; JNC) were used to fill the cells. The LC was injected using a syringe through capillary force, and the cell gap was controlled to a uniform 60 μm. POM (BXP 51, Olympus, Tokyo, Japan) was employed to assess the molecular alignment. The electrical properties of the Ag-doped NiO alignment layer were studied using AP LC cells with a 5 μm uniform cell gap. The polar anchoring energy of the LCs on the films was measured over a voltage range of 0–20 V, and capacitance–voltage curves were obtained to investigate the hysteresis characteristics (LCR meter, Agilent 4284A, Santa Clara, CA, USA).

## 3. Results and Discussion

**Figure 1:**
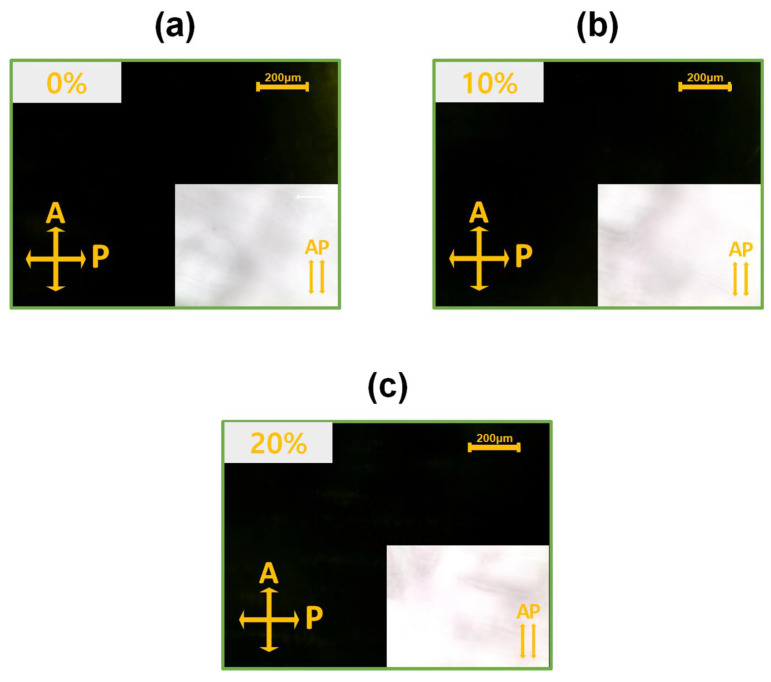
Polarized optical microscopy images of AP LC cells fabricated using nickel oxide films doped with (**a**) 0, (**b**) 10, and (**c**) 20 wt% Ag. The yellow arrows indicate the directions of the analyzer (A) and polarizer (P).

To examine the alignment of LCs on Ag-doped NiO films, AP LC cells were fabricated using NiO films doped with 0, 10, and 20 wt% Ag. The LC cells were analyzed using POM, and the results are shown in Figure 1. When the polarizer and analyzer were oriented perpendicular to each other, the LC cells displayed a dark, uniform image, indicating that the LCs were aligned consistently across the film. When light passed through the AP LC cells, the uniformly aligned LCs guided the light in a single direction. Therefore, when the polarizer and analyzer above and below the LC cell are perpendicular to each other, the POM measurements represent a dark image (indicating no light leakage and perfect light blocking. Conversely, when the polarizer and analyzer were parallel, light passed through the LC cell, resulting in a bright white image, as shown in Figure 1. This demonstrates that the LC molecules are uniformly aligned, as confirmed by the POM results.

**Figure 2:**
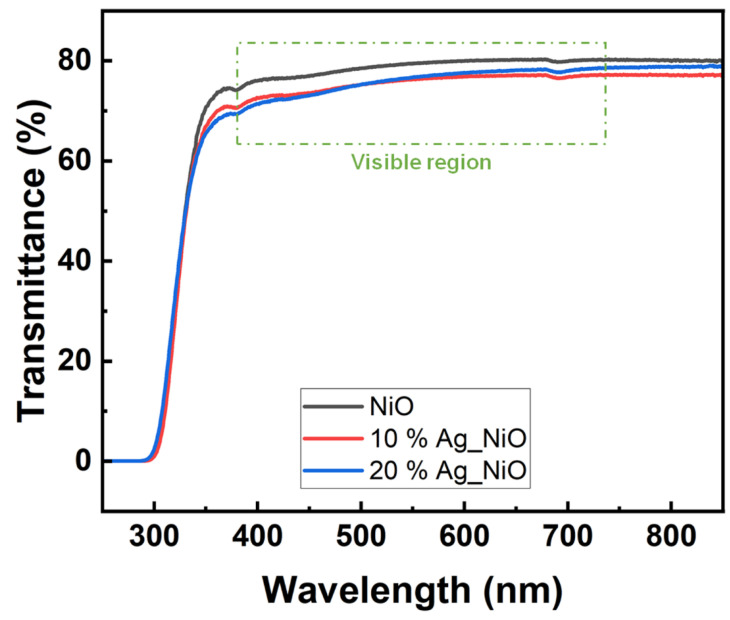
Transmittance spectra of nickel oxide films doped with 0, 10, and 20 wt% Ag.

The optical transmittances of the brush-coated Ag-doped NiO films, with doping concentrations of 0, 10, and 20 wt%, were measured in the 250–850 nm wavelength range, as shown in Figure 2. The transmittance spectra for these films displayed near-horizontal lines in the visible region (380–740 nm), indicating stable optical transmittance. The average optical transmittances in the visible region were found to be 78.83%, 75.59%, and 75.91% for the 0, 10, and 20 wt% Ag-doped NiO films, respectively. Since nickel (II) chloride, the precursor used, is green in color, the resulting NiO films initially showed low optical transmittance. However, the average transmittance of the uncoated glass and indium-tin-oxide-coated glass substrates were 85.4% and 81.8%, respectively, suggesting that Ag-doped NiO films are suitable for use in LC electronic devices.

**Figure 3:**
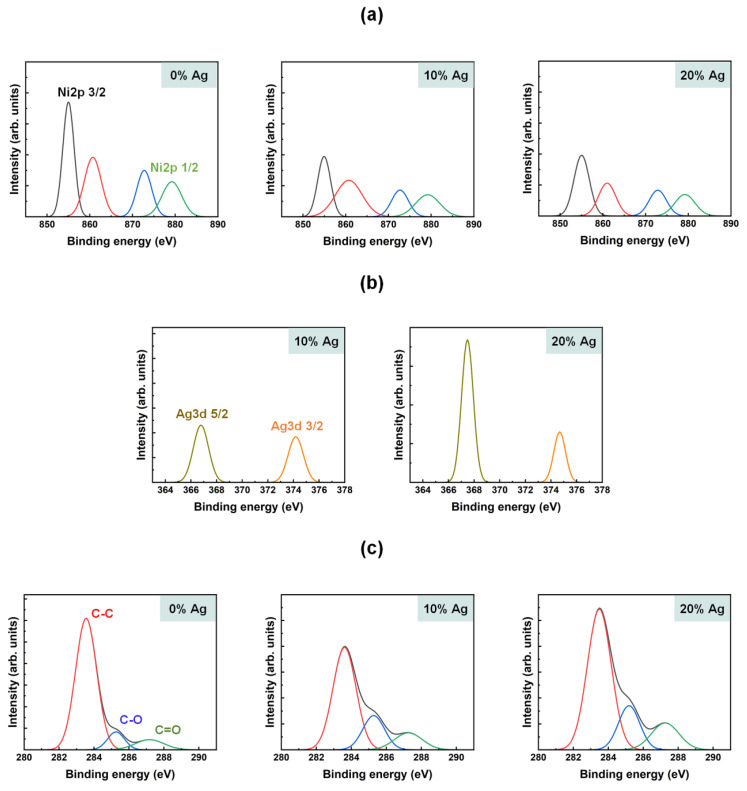
X-ray photoelectron spectra of nickel oxide films doped with 0, 10, and 20 wt% Ag: (**a**) Ni 2p, (**b**) Ag 3d, and (**c**) C 1s core levels.

The chemical composition of the brush-coated NiO films, doped with 0, 10, and 20 wt% Ag, was analyzed using XPS, as shown in Figure 3. The Ni 2p (Figure 3a), Ag 3d (Figure 3b), and C 1s (Figure 3c) core-level spectra were measured, revealing the main components of the Ag-doped NiO films. In the Ni 2p spectra, four sub-peaks were observed, corresponding to Ni 2p3/2, Ni 2p1/2, and two satellite peaks. The 3/2 and 1/2 peaks were centered at 854.5–855.5 eV and 872.0–873.0 eV, respectively, and the intensities of these peaks decreased as the Ag doping concentration increased. In contrast, the intensity of the Ag 3d spectra increased with higher Ag concentrations. These spectra displayed two sub-peaks corresponding to the 5/2 and 3/2 states, with binding energies centered at 366.5–367.5 and 374.0–375.0 eV, respectively. The C 1s spectra were deconvolved into three sub-peaks, representing carbon single bonds, carbon–oxide–metal bonds, and carbon double bonds. The corresponding binding energies were centered at 283.0–284.0, 285.0–286.0, and 287.0–288.0 eV, respectively. The intensity of the carbon–oxide–metal bond peaks increased as the Ag doping concentration increased, confirming the successful incorporation of Ag into the NiO films.

**Figure 4:**
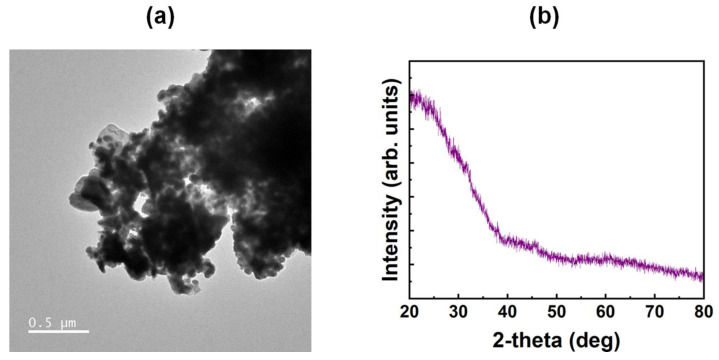
(**a**) Transmission electron microscopy image and (**b**) X-ray diffraction pattern of the 20 wt% Ag-doped nickel oxide film.

The surface morphology of the Ag-doped NiO films containing Ag nanoparticles was investigated using TEM, as shown in Figure 4a. The TEM images revealed agglomerates of NiO particles along with dispersed spherical Ag nanoparticles, confirming the formation of the Ag-doped NiO films. The crystalline properties of the films were further examined using XRD analysis, shown in Figure 4b. The XRD spectra, measured in the 20–80° angular range, did not show any prominent peaks, indicating that the Ag-doped NiO films possess an amorphous structure. This is consistent with metal-oxide films formed via solution processing, which typically exhibit amorphous characteristics at curing temperatures below 500 °C [27]. The absence of crystalline peaks further suggests that the amorphous structure does not hinder the uniform molecular alignment of the LCs on the Ag-doped NiO film surface.

**Figure 5:**
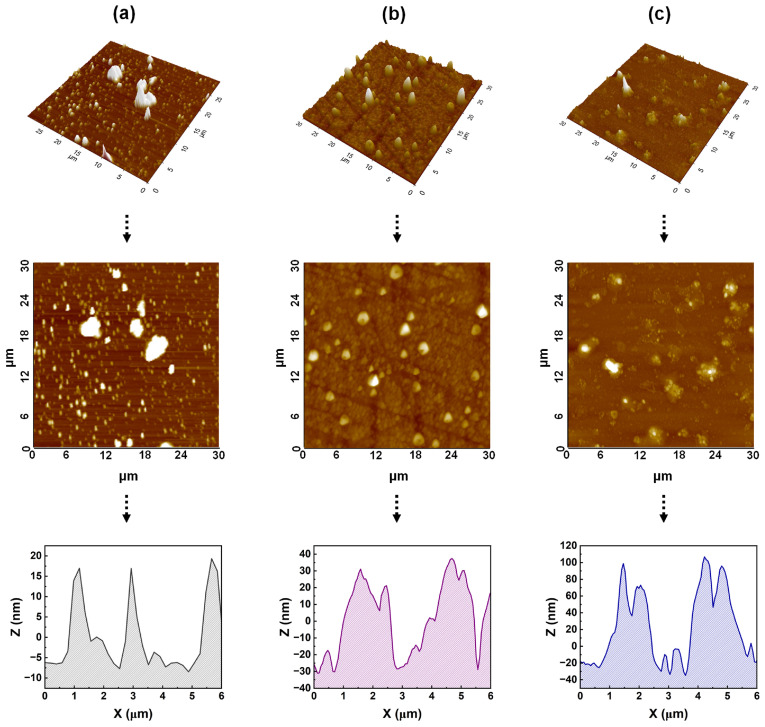
Atomic force microscopy images and corresponding line profiles of nickel oxide films doped with (**a**) 0, (**b**) 10, and (**c**) 20 wt% Ag.

The surface morphology of the Ag-doped NiO films was further characterized using AFM, as shown in Figure 5. AFM images revealed lumps on the surface of the pure NiO film, likely due to the condensation of NiO sol particles during the brush coating process. At Ag doping concentrations of 10 and 20 wt%, small topographic structures were observed on the surface. This is attributed to the effects of the brush coating process, the dispersion of Ag nanoparticles, and the formation of the NiO films. The arithmetic average roughness values were 11.7, 28.3, and 22.0 for the 0, 10, and 20 wt% Ag-doped NiO films, respectively. The corresponding line-profile data also indicated the presence of nano- and micro-groove structures on the film surface, which were a result of the shear stress induced by the brush hair movement during coating [28,29]. The unidirectional coating and brush-hair movement led to the anisotropic distribution of the NiO sol, which, upon curing, transformed the sol into a gel with incorporated Ag nanoparticles, resulting in an anisotropic film surface. This anisotropic surface imposes geometric constraints on the LC molecules, promoting uniform molecular alignment, as observed in the POM analysis [30,31,32,33].

**Figure 6:**
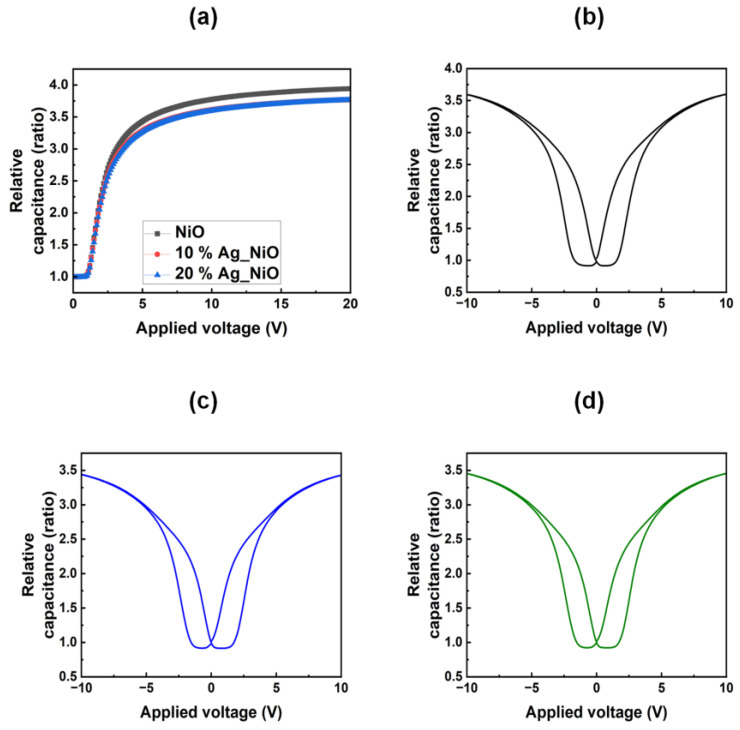
(**a**) Polar anchoring energy curves of LC cells measured from nickel oxide films doped with 0, 10, and 20 wt% Ag. Hysteresis properties of LC cells assembled with (**b**) 0, (**c**) 10, and (**d**) 20 wt% Ag-doped nickel oxide films.

The alignment and electrical stability of the LC molecules on the Ag-doped NiO films were further investigated by measuring the polar anchoring energy. The corresponding data are shown in Figure 6a. The polar anchoring energies were calculated to be 3.05 × 10^−4^, 2.65 × 10^−3^, and 3.84 × 10^−3^ J m^−2^ for the 0, 10, and 20 wt% Ag-doped NiO films, respectively. These results demonstrate that the electrical stability of the Ag-doped NiO films improves as the Ag doping concentration increases. The hysteresis behavior, which is indicative of image sticking in LC electronic devices, was also investigated for the 0 (Figure 6b), 10 (Figure 6c), and 20 (Figure 6d) wt% Ag-doped NiO film-based LC cells. The residual DC voltage, which influences the hysteresis effect, was found to be 1.660, 1.643, and 1.621 V for the 0, 10, and 20 wt% Ag-doped NiO films, respectively. These results suggest that Ag doping improves the display stability by reducing afterimage effects.

## 4. Conclusions

This study successfully fabricated Ag-doped NiO films through a solution-based process, utilizing them as a uniform alignment layer for LC molecules. The Ag doping concentrations were precisely controlled at 0, 10, and 20 wt%. XPS analysis confirmed that the films were well-formed as intended. AFM analysis revealed a nano/microgroove structure, with anisotropic properties resulting from the motion of brush hairs during the solution coating process. XRD analysis indicated that the films possessed an amorphous structure, while their optical transmittance was found to be suitable for use in electronic display devices. POM analysis demonstrated the films’ ability to achieve uniform LC molecular alignment and effective optical control. Additionally, Ag doping enhanced both the polar anchoring energy and the stability of the LC alignment layer. The polar anchoring energy increased by 1159.02% after Ag doping. The films also exhibited reduced hysteresis characteristics and improved resistance to image sticking, as evidenced by the decrease in residual charge. Overall, these findings highlight the potential of Ag-doped NiO films for use in electronic display applications.

## Data Availability

The data presented in this study are available upon request from the corresponding author.
